# Harnessing health information technology to promote equitable care for patients with limited English proficiency and complex care needs

**DOI:** 10.1186/s13063-024-08254-y

**Published:** 2024-07-04

**Authors:** Inna Strechen, Patrick Wilson, Targ Eltalhi, Kimberly Piche, Dan Tschida-Reuter, Diane Howard, Bruce Sutor, Ing Tiong, Svetlana Herasevich, Brian Pickering, Amelia Barwise

**Affiliations:** 1 Department of Anesthesiology and Perioperative Medicine, Division of Critical Care, Mayo Clinic, Rochester, MN USA; 2 Kern Center for the Science of Health Care Delivery, Mayo Clinic, Rochester, MN USA; 3 Language Services, Mayo Clinic, Rochester, MN USA; 4 Language Services Operations Manager, Mayo Clinic, Rochester, MN USA; 5 Language Services Operations Administrator, Mayo Clinic, Rochester, MN USA; 6 Department of Psychiatry and Psychology and Medical Director of Language Services, Mayo Clinic, Rochester, MN USA; 7 Information Technology, Mayo Clinic, Rochester, MN USA; 8 Biomedical Ethics Research Program and Division of Pulmonary and Critical Care Medicine, Mayo Clinic, Rochester, MN USA

**Keywords:** Healthcare disparities, Non-English language preference (NELP), Complex care needs, Language services, Complexity score, AI, In-person interpreter

## Abstract

**Background:**

Patients with language barriers encounter healthcare disparities, which may be alleviated by leveraging interpreter skills to reduce cultural, language, and literacy barriers through improved bidirectional communication. Evidence supports the use of in-person interpreters, especially for interactions involving patients with complex care needs. Unfortunately, due to interpreter shortages and clinician underuse of interpreters, patients with language barriers frequently do not get the language services they need or are entitled to. Health information technologies (HIT), including artificial intelligence (AI), have the potential to streamline processes, prompt clinicians to utilize in-person interpreters, and support prioritization.

**Methods:**

From May 1, 2023, to June 21, 2024, a single-center stepped wedge cluster randomized trial will be conducted within 35 units of Saint Marys Hospital & Methodist Hospital at Mayo Clinic in Rochester, Minnesota. The units include medical, surgical, trauma, and mixed ICUs and hospital floors that admit acute medical and surgical care patients as well as the emergency department (ED). The transitions between study phases will be initiated at 60-day intervals resulting in a 12-month study period. Units in the control group will receive standard care and rely on clinician initiative to request interpreter services. In the intervention group, the study team will generate a daily list of adult inpatients with language barriers, order the list based on their complexity scores (from highest to lowest), and share it with interpreter services, who will send a secure chat message to the bedside nurse. This engagement will be triggered by a predictive machine-learning algorithm based on a palliative care score, supplemented by other predictors of complexity including length of stay and level of care as well as procedures, events, and clinical notes.

**Discussion:**

This pragmatic clinical trial approach will integrate a predictive machine-learning algorithm into a workflow process and evaluate the effectiveness of the intervention. We will compare the use of in-person interpreters and time to first interpreter use between the control and intervention groups.

**Trial registration:**

NCT05860777. May 16, 2023.

**Supplementary Information:**

The online version contains supplementary material available at 10.1186/s13063-024-08254-y.

## Background and significance

The United States healthcare system continues to face persistent challenges in providing medical care to an increasingly diverse and multilingual patient population [1–3]. Optimal approaches to providing sufficient and high-quality interpretation remains challenging [4–6]. Studies indicate significant disparities in the quality of care for individuals who have a non-English language preference (NELP) and complex medical conditions [7–17]. When admitted to the intensive care unit (ICU), those patients become particularly vulnerable to an increased risk of medical errors and may experience less favorable outcomes compared to their English-speaking counterparts [8, 18–20]. These disparities include prolonged ICU stays, a higher likelihood of ICU mortality, increased utilization of aggressive interventions, and suboptimal symptom management [9, 10, 20]. While the literature underscores the benefits of professional interpretation, clinicians’ inconsistent engagement with such services may lead to patients with NELP not receiving the entitled and essential interpretation they need [21–32].

Clinical encounters in the ICU often involve significant educational or psychosocial components, demanding nuanced communication where in-person interpretation can be more effective [19, 33–39]. To save time and potentially because of concerns about cost to the patients, clinicians may avoid engaging interpreters [19, 40–42]. Moreover, the COVID-19 pandemic has reduced the availability of in-person professional interpreters and increased the use of remote phone and video interpretation [43, 44]. While these remote options have been deployed to maintain healthcare services, they cannot always foster the desired inclusivity, particularly for interactions involving patients with complex care needs [44, 45]. In-person interpreting offers significant potential in addressing healthcare disparities and accurately interpreting non-verbal cues among patients facing critical and complex illnesses where precise understanding is vital [33]. In-person interpreters can provide a deeper understanding of cultural considerations, enhancing the quality of communication and ensuring that every word and sentiment is understood as intended [33, 39].

There is a substantial knowledge gap regarding how to prioritize patients for in-person professional interpreters in settings where there is a shortage of in-person interpreters and high reliance on ad hoc or virtual interpretation [46]. These factors present significant difficulties in addressing the needs of inpatients with NELP and complex healthcare requirements [26]. Integrated health information technology (HIT) and analytic solutions that develop mechanisms to prioritize services and streamline the process for engaging in-person interpreters may be helpful for addressing this shortage [30, 47–54].

### Language and medical complexity risk scores

In order for healthcare organizations and clinicians to address the language, cultural, and health literacy barriers of inpatients with NELP and complex healthcare needs, it is crucial for them to identify the patients most likely to benefit from language assistance and prioritize them. Although guidance exists for evaluating if a person has language proficiency, healthcare systems do not routinely assess proficiency [55–57]. We encourage healthcare facilities to adopt a consistent method for identifying patients with language barriers and complex medical needs, and the algorithm to be tested in this study might fulfill this proposed standard. We believe that this combined with proactive outreach efforts will also encourage increased use of in-person interpreters, ultimately addressing healthcare disparities and improving the overall quality of care.

With the protocol described in this paper, we plan to test and evaluate the impact of an artificial intelligence enabled intervention on the identification of patients with complex care needs and language barriers and subsequent in-person interpreter service utilization. The system, known as Control Tower (CT), is a fully integrated information technology (IT) system solution which pulls and processes medical data presenting the results through an ordered patient list in a custom graphical user interface (GUI). The algorithm extracts information from electronic health records (EHR)s, predicts complex care needs to stratify those who would benefit from an in-person interpreter, and allows a human operator to review the predictions and provide their evaluation to language services. The following integration phase into the workflow entails notifying the clinical team about identified patient needs and facilitating a process to connect interpreters with patients and clinicians.

### Development of integrated language complexity score algorithm

Building on our current integrated palliative risk score algorithm that uses machine learning predictive analytics for defining need for palliative care as well as EHR data—such as length of stay, events list (clinical notes from teams and services involved in care, procedural notes and diagnostic reports), and level of care (ICU/PCU/floor)—we have developed a complexity score [58, 59]. This score is combined with informatics data about the patient (“preferred language not English”) in the EHR to identify those who would benefit from an in-person interpreter. The algorithm, along with other contextual patient data, is integrated into a graphical user interface (GUI) that allows a human operator, known as the Control Tower Operator (CTO), to review and validate its forecast.

### Evaluation of artificial intelligence (AI)

This paper describes work we will do to implement a complexity score among inpatients with NELP to identify those that would benefit from an in-person interpreter. We consider patients with complex care needs as those with a high burden of disease, those experiencing critical or serious illness, those with a life-limiting illness, and those with palliative care needs. To our knowledge, this type of score—combining NELP and complexity—has not been previously developed or implemented in practice. We have conducted other work as a foundation for the trial to ascertain the perceived risks and benefits of AI to improve in-person interpreter use [60]. Successful integration into the practice workflow is key for AI to be useful in clinical care [61–65]. Algorithms cannot earn trust solely through research showcasing predictive performance (phase I) or clinical assessment (phase II). It requires two additional phases. The first involves field testing through clinical trials to showcase an impact on clinical outcomes (phase III), alongside establishing an infrastructure for prospective monitoring during routine usage [66].

### Aims of the study

The aim of this study is to assess the effectiveness of our comprehensive intervention, integrating artificial intelligence (AI) with a human operator into the language services process to provide in-person interpreters to patients with complex care needs. The study will focus on evaluating the impact of the combined human-AI intervention, along with the defined process (active outreach to healthcare teams caring for identified patients) on in-person interpreter utilization in the target population. *Hypothesis statement*: By employing a machine learning algorithm to identify individuals with NELP and complex care needs, patients will be more likely to receive an in-person interpreter and at an earlier stage of the hospitalization compared to standard care.

### Trial design

To achieve the objectives, we will conduct a two-armed stepped wedge cluster randomized trial in both inpatient campuses at Mayo Clinic, Rochester. Trial units will undergo a HIT assessment of patients who would benefit from in-person interpreter support, allowing a human operator (CTO) to validate the forecast and notify the language services about the identified patient needs. Patients in control units will receive usual standard of care relying on their clinicians to reach out to interpreter services to request an in-person interpreter or use another type of interpretation modality such as video or phone.

## Methods/design

### Study setting

From May 1, 2023, to June 21, 2024, a single-center stepped wedge cluster randomized trial will be conducted within 35 units of Saint Marys Hospital and Methodist Hospital at Mayo Clinic in Rochester, Minnesota. The units include medical, surgical, trauma, and mixed ICUs and hospital floors that admit acute medical and surgical care patients as well as the emergency department (ED). The transitions between study phases will be initiated at 60-day intervals resulting in a 12-month study period. Mayo Clinic’s Institutional Review Board (IRB) approved the study as minimal risk (IRB-22-002974) and waived the requirement to obtain individual patient and clinician and provider consent due to the pragmatic nature of the design. The study has been registered on ClinicalTrials.gov NCT05860777.

### Eligibility criteria

The recruitment and enrollment processes are broad and designed to simulate the use of the CT in practice. We will include those patients who will be admitted to the hospital or seen in the ED during the study period. Patients who have a NELP and complex care needs identified by the algorithm will be eligible. NELP will be identified and confirmed in several ways: through the algorithm-generated reports as well as using manual confirmation in the EHR by a human operator. Complexity will be identified using a palliative care score and supplemented by other predictors of complexity, such as length of stay, level of care, procedures, events, and clinical notes.

Patients will be excluded from the review if they are less than 18 years; do not have a language listed in EHR or any evidence of interpreter use; use sign language; are a confidential, unidentified, or non-verbal patient; or have an incomplete EHR. Patients who do not authorize the use of their EHR for research in accordance with the Minnesota state statute will be also excluded from the study [67].

### Intervention

**Fig. 1:**
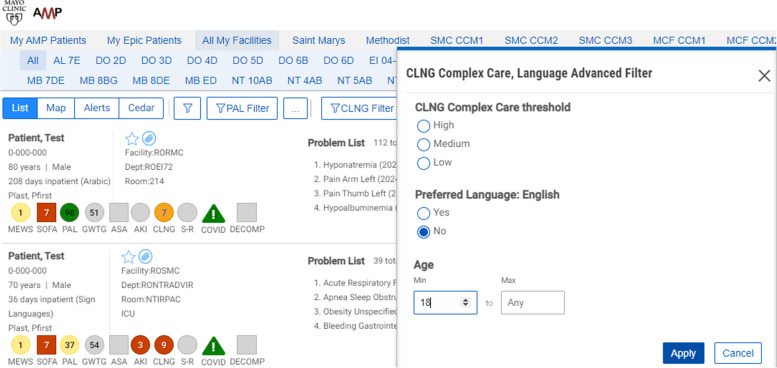
Screenshot of the Control Tower user interface

This study will be conducted using a workstation and software tool known as Control Tower. The CT is a web browser application that extracts medical data, processes the prediction algorithm, and presents the results through an ordered patient report list. See Fig. 1. More details can be found in Murphree et al.’s work [68].

In addition to the complexity score, additional data on language needed, age, other common risk scores, hospital unit/floor, and current length of stay are available and presented in the report to provide context for the calculated complexity score.

Patients with NELP in the intervention units will have complexity scores calculated within the Control Tower (≥ 9 points = severe complexity; 4–8 points = moderate complexity; < 4 points = mild complexity). Higher scores indicate increased complexity and need for in-person interpreter. Patients with NELP and calculated complexity scores are subsequently ranked from highest to lowest complexity score, as well as being color coded, with red indicating a score of 9, orange ≤ 9 ≥ 4, and yellow < 4. Newly admitted patients who are currently being evaluated by the algorithm have their scores labeled as grey. The complexity score is based on a machine learning palliative care score, supplemented by other predictors of complexity, including length of stay and level of care as well as events (clinical notes from teams and services involved in care, procedural notes, and diagnostic reports) (see Supplemental Table S1).

As part of the intervention, a CTO will engage with the language services coordinator responsible for coordinating, organizing, and providing in-person interpreters at study sites. The CTO will monitor the CT during regular weekdays starting early in the morning and generate a list once daily at 7 am. This list comprises adult patients with NELP and complex care needs, ordered by their complexity scores (from highest to lowest), and is assessed for any additional exclusion criteria in the development of the final list. Upon completing the screening process, the CTO will send the ordered list to the language services coordinator.

Units in the stepped wedge design will be randomized in their order for receiving treatment by a random sorting algorithm with a set seed for reproducibility. For our data pipeline, all dates for transition will be hard-coded so that the study participants enter the list when their admission corresponds to a study wedge. At each wedge transition, study staff will review the list to ensure that the program correctly switches patients over at the appropriate time. The number of patients on the list will vary and increase as we move forward with each stepped wedge of the trial every 60 days (adding 7 units to the intervention arm with each wedge). However, by working closely with language services leadership and operations managers, we believe this will be feasible even as the numbers in the intervention group grow. Rather than limiting the number of patients eligible to be included on the list sent to language services, it was agreed that should the list of patients in the intervention group become too large for disseminating secure chat messages and providing in-person interpreters, the organized list would help language services prioritize those patients with the highest complexity scores. This allows for the matching of patient need with the expected capacity of the language services team which is a pragmatic approach to balance effectiveness while avoiding interruptions to the usual workflow throughout the trial.

For those patients who are in the intervention arm, the language services coordinator will record if they have in-person interpreter for that particular language. Then, the language services coordinator will send a secure chat message via the EHR to advise the bedside nurse that the patient would benefit from an in-person interpreter and include contact information for how to reach language services. The secure chat message will be “Good morning. For an in-person interpreter, please contact Language Services at ext. X-XXXX with a date & time. We will do our best to meet the patient’s and medical team’s needs”. In contrast, the patients in the control units will continue to receive the regular standard of care, with patients potentially receiving an in-person interpreter or another interpretation modality following standard procedures by the primary healthcare team. Those patients in the control group will rely on clinician initiative to request language services.

Due to the potential disruption of using a new approach and tool such as a complexity score and CTO for promoting in-person interpreter use, we have communicated with multiple practice leaders and divisional and departmental committees. Additionally, we plan to send email communications to unit nurse managers prior to each stepped wedge roll out to prepare them.

In our study, 68% of the in-person interpreters are certified at the highest level for their respective languages by the Multiple National Certifying Organizations for Medical Interpreters. The remaining staff are in the process of certification, adhering to a strict code of ethics and standardized testing to ensure proficient language skills. Both study arms receive the same interpretation personnel and quality, but they follow different processes to access language services.

### Outcomes

For all study outcomes, data will be collected through either the EHR or language services report list. The data outcomes will be abstracted during the patient hospitalization. The primary outcome is the number of patients with NELP and complex care needs who use an in-person interpreter during hospitalization in the units of interest as measured by language services daily report list. The secondary outcome will be time to first use of in-person interpreter—measured as time in hours and minutes from admission to in-person interpreter use as measured by the language services team electronic documentation system. The secure chat process measures including if sent, if responded to, and if not sent the reason such as no in-person interpreter on staff or available that day will be collected daily.

### Participant timeline

**Fig. 2:**
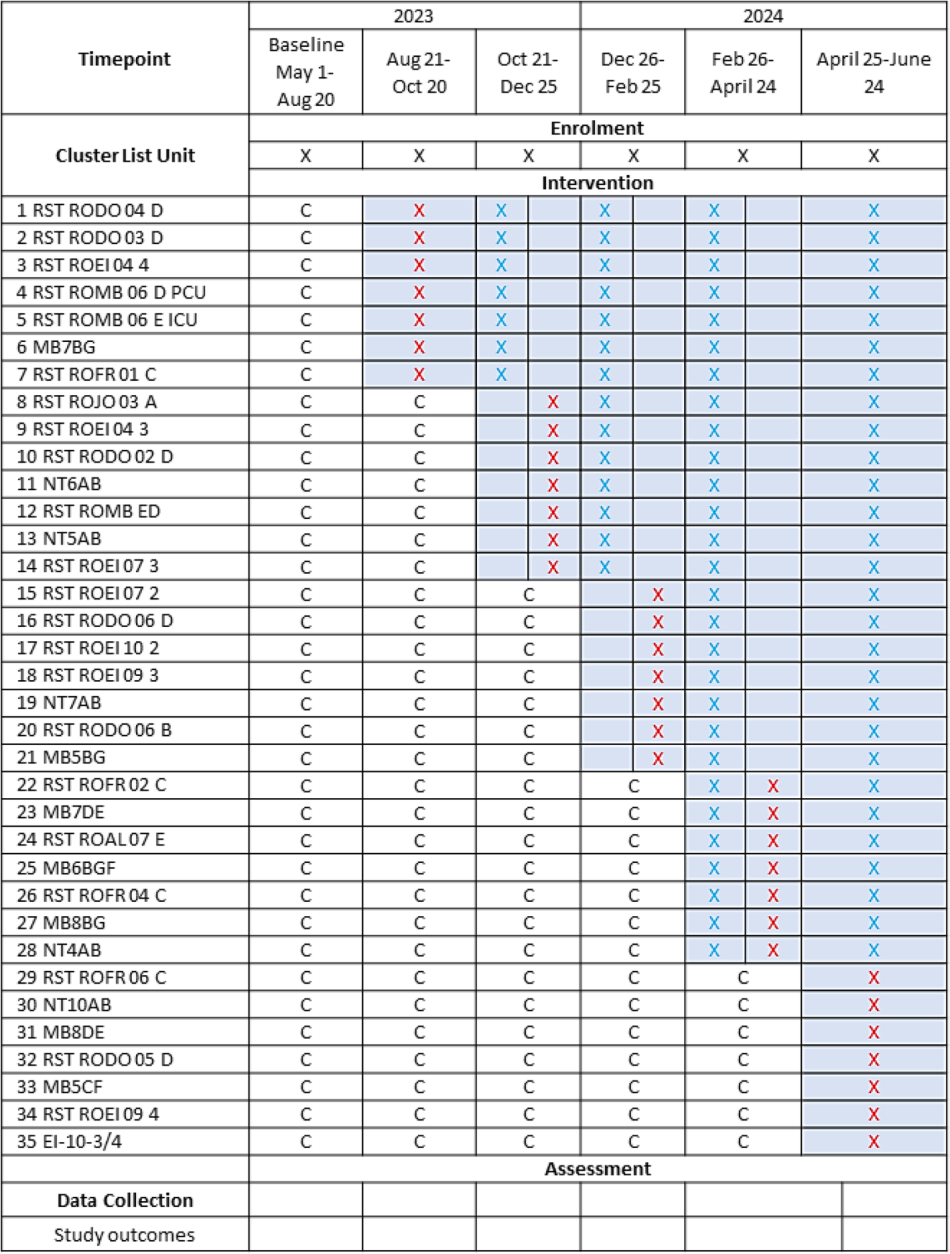
SPIRIT figure—stepped wedge cluster randomized study design. The trial will be conducted over 12 months with 12 inpatient units crossing from control to intervention  in 60-day steps. Usual care is denoted by “c”

The stepped wedge design involves allocating 35 floor and ICU units into a design matrix comprising 5 treatment wedges. Computer allocation will be used to generate the allocations prior to the start of the study. Each unit will cross over randomly from the control group (standard clinical practice and care) to the intervention group. Each wedge will span approximately 60 days, resulting in a study period of approximately 12 months unless specified otherwise. The initial step will entail a baseline period during which no intervention is administered, with all clusters receiving the intervention in the final step. Due to the pragmatic design of the trial, clinicians cannot be blinded to patient allocation to the intervention or control units, and we do not plan to implement blinding during the analysis as our endpoints are objective, and diffused roll-out would make this impossible [69]. This is most relevant if there are patient-reported outcomes. Patients will receive standard in-person interpreter services if requested and available in the hospital; however, those in the intervention group will differ in that clinicians will have been alerted to the patient’s need based on complexity score, CTO review and filter and secure chat sent by language services personnel. No additional patient data beyond the hospitalization will be collected, and there will be no follow-up visits. See Fig. 2.

### Data analysis plan

#### Power statement

The proposed investigation will use a stepped wedge cluster randomized design with 12 clusters. Based on preliminary data, it is estimated that there are > 9000 inpatient admissions with NELP annually at our institution of which only 14 to 16% use the services of an in-person interpreter at some point during their hospitalization. For sample-size/statistical power considerations, we assume that the proportion of patients with NELP receiving in-person interpreter services at the start of our study period (while all patients are receiving usual care) will be 0.15. Although not all inpatients with NELP will meet study inclusion criteria, we believe that over the course of the year, the total number of inpatients who meet study inclusion criteria will be in the range of 500 to 700 for each of the 12 clusters. Stepped wedge cluster randomization trials typically have more statistical power than other cluster randomized designs when clusters are correlated, because each cluster is able to serve as its own control. Within-cluster correlation was introduced by using cluster-specific baseline rates (which ranged between 10 and 20%), and within-cluster correlation coefficients were not specified. Because of the complex nature of the design, we estimated statistical power using Monte Carlo simulation [70]. The Monte Carlo simulation for this paper was generated using the SAS software (Copyright © [2024] SAS Institute Inc. SAS and all other SAS Institute Inc. product or service names are registered trademarks or trademarks of SAS Institute Inc., Cary, NC, USA.) A logistic regression model was used for the simulation with the outcome being the use of in-person interpreter services. Simulations were created with the overall baseline percentage of patients receiving in-person interpreter services set at 15% (with cluster-specific baseline rates set between 10 and 20%) and included slight upward secular trend over time. Supplemental Table S2 presents the statistical power provided for detecting potential intervention effects corresponding to odds ratios ranging from 1.35 to 1.65 using 3 various sample size scenarios (500, 600, and 700 patients per cluster). Based on these simulations, the proposed investigation should have statistical power of > 80% to detect an odds ratio of 1.5 or greater.

We will generate overall and cluster-specific summaries of patient characteristics (sex, age, primary language, risk scores, etc.) using descriptive statistics, including mean ± SD for continuous variables with frequencies and percentages for nominal variables. The entire study population will be analyzed following an intention-to-treat (ITT) approach. The ITT analysis will include all patients in the intervention group regardless of whether the secure chat was sent and regardless of whether the clinician requested the service after receiving the secure chat. This principle will be extended to the cluster status in the event of transfers between intervention and control units. All missing data will be analyzed using complete case analysis. The primary outcome will be a binary variable representing any use of interpreter services during hospitalization. To assess the effects of the intervention, the primary outcome will be analyzed using a generalized linear mixed-effects model with variables: intervention approach and time period. The inpatient unit will be denoted as the random effect in the model to characterize the correlation among patients within the same cluster. An additional secondary outcome will be evaluated as the time from “intervention” to first use of an interpreter. Eligible patients are identified at 7 am daily, Monday to Friday. Time of intervention is calculated from the first time a patient is identified until documentation of first use of an interpreter. Patients may be eligible on multiple days and calculation of this outcome will start evaluation at 7 am on the first eligible day identified by the CTO. Those who never receive an interpreter will be assigned an adverse value (an arbitrarily large amount of time, reflecting an outcome worse than any observed time to interpreter). A generalized linear mixed-effects model with proportional odds link function will be used to evaluate this outcome to account for the stepped wedge cluster randomized study design including adjustment for time period and random effect for cluster. An alternative approach may consider reporting cumulative incidence estimates of time to interpreter by intervention group, with death or discharge a competing risk, which is functionally similar to the assignment of large arbitrary value for those without interpreter services. Patients who received interpreter services prior to identification by the CTO will be excluded from analyses as they do not meet inclusion criteria at “time zero” or baseline of the analysis. In all cases, the intervention effect will be summarized by reporting point estimates and corresponding 95% confidence intervals. Two-tailed *p*-values < 0.05 will be considered statistically significant [71, 72].

### Data management

All data pertaining to study outcomes/model covariates and process measures will be collected through the following methods: (1) all input data received from the machine learning model will be logged each time the algorithm is called and stored in a study database; (2) study outcomes will be collected from the hospital EHR and language services daily report; (3) process measures (such as the number of secure chat messages sent and reasons not sent or in-person interpreter declined) will be gathered through the daily logs exchanged between the CTO and the language services team.

### Data monitoring

The proposed intervention has been reviewed by the IRB and was determined to be a minimal risk study, so no data monitoring committee (DMC) will be created. Consequently, there will be no interim analyses or predefined stopping rules for prematurely ending the trial. The anticipated risks to patients in this study are expected to align with those encountered in routine clinical care. Ensuring patient safety will primarily rely on clinical staff adhering to established standards of care. Study logs will undergo bi-monthly audits for reporting purposes, but no decisions will be made based on the data to either stop or continue the trial.

The evaluation of study logs will involve several diverse personnel, the CTO, PI, study team statistical analyst, and IT personnel. The interpreter services personnel and CTO will monitor the algorithm while utilizing the tool to identify any potential errors affecting their workflow, such as missing complexity scores or inaccuracies in data elements or score components. The study team, PI, statistical expert, and IT support will review the logs to ensure complete field entries and mitigate omissions. The IT study team members will oversee the data pipelines to guarantee proper functionality across all data systems involved in score calculation. We will document all days when the pipeline fails to fire.

### Confidentiality

Patient participation will occur through the utilization of hospital interpreter services with no additional contact or visits needed; therefore, we will follow the hospital’s policies and procedures for maintaining patient privacy and confidentiality with respect to data. For report purposes, we will use Agency for Healthcare Research and Quality (AHRQ) guidelines [73]. All results will be reported in aggregate with no cells size smaller than 10.

### Dissemination policy

We anticipate given the novelty of our proposed work that we will have an opportunity to publish in the scientific literature regardless of outcome. Trial summary results will be submitted to ClinicalTrials.gov following the completion of the trial. The team have experience with publication, and we will follow all standard authorship and ethical requirements as specified in journals in which we publish. We anticipate that all authors of this protocol paper will also be authors of the subsequent outcome paper. We will use the Equator guidelines for reporting of pragmatic clinical trials to report our results [74]. Furthermore, here, we have included a checklist of recommended items to provide in clinical trial protocol [75] (Supplemental Table S3).

## Discussion

This research explores the impact of incorporating a ML algorithm into a healthcare system to facilitate timely and more frequent in-person interpreter services for inpatients with NELP and complex medical needs. There are substantial gaps in our understanding of how these algorithms operate in real-world clinical settings due to the challenges with integration and evaluation. Aside from understanding the perceived risks and benefits of using AI in this domain, for AI to benefit patients, effective integration into clinical practice workflows is essential [60, 64, 68]. This work does not delve into specific implementation issues, unlike some other studies [62]. Additionally, akin to any clinical intervention, evaluating a predictive model, its deployment, and its impact should entail robust assessment beyond merely examining predictive accuracy and reliability [61, 63]. Developing an algorithm as well as the required infrastructure for real-time implementation and devising a workflow model that seamlessly integrates the algorithm into clinical practice demands interdisciplinary teamwork. The diverse IT skills and expertise needed as well as the collaboration with leadership and clinical groups can be challenging and needs sustained effort for success.

We should also note that this research project and protocol benefits from previous work several members of our broader research study team conducted developing a palliative care algorithm to increase palliative care referrals for patients who needed it based on ML predictive analytics [58, 68]. Based on the significantly improved patient outcomes such as increased use of palliative care and reduced readmissions, that algorithm has since been incorporated into routine clinical care throughout much of the broader hospital and enterprise beyond where it was initially developed and tested. It has also been updated and will be a component of the complexity score that will be used in our trial.

This pragmatic clinical trial design should be considered in light of noteworthy strengths and limitations. This study design offers various benefits, such as reducing contamination among clinicians by implementing interventions at the unit level, mirroring how organizations typically introduce interventions gradually in real-world settings, and requiring fewer clusters, thus ensuring feasibility [71, 76, 77]. Given its integration into clinical practice across all acute care units in both Rochester campuses, the trial boasts a well-represented patient population with minimal exclusion criteria and waivers for both patient and clinician consent. Furthermore, it imposes minimal burden on patients or clinicians as there are no complex study visits, procedures, or evaluation questionnaires to be completed. All assessments will be conducted by the research team and are inherent to the healthcare model, designed to complement usual care without causing disruption.

Despite these strengths, pragmatic clinical trials such as this also have some drawbacks. We are relying on routinely collected data from language services. Other important outcome measures such as satisfaction with care and decision making as well as healthcare utilization would be challenging to ascertain. In addition, the study was powered for our chosen outcome measures. Finally, the description of this protocol and submission for publication was somewhat delayed and ideally would have occurred earlier during the overall study timeline. Several factors contributed to the delay in protocol submission: team members working part-time, pursuing further education, and preparing for exams; the necessity of coordinating feedback from multiple authors across different teams, which required extra time to ensure a thorough review and consensus; competing priorities among the investigators involved; and our commitment to maintaining the standard of care provided by language services without any disruption.

## Trial status

The protocol submitted here is version 1.1 dated February 23, 2024. The study began on August 21, 2023. Recruitment for the study commenced on May 1, 2023, and is expected to continue until June 21, 2024.

### Supplementary Information

Supplementary Material 1: Supplemental Table S1. Complexity score. Supplemental Table S2. Statistical power to detect the given intervention effect 3 sample-size scenarios. Statistical power was estimated with Monte Carlo simulation assuming that the overall baseline percentage of patients received interpreter services is 15% and cluster specific percentages range from 10 to 20%. Supplemental Table S3. WHO Trial Registration Data Set information (Version 1.3.1).

Supplementary Material 2.

## Data Availability

Data is available upon reasonable request.
