# Supplementary material for: Harnessing health information technology to promote equitable care for patients with limited English proficiency and complex care needs
Source: Trials. 2024 Jul 4;25:450. doi: 10.1186/s13063-024-08254-y (PMC11223355; doi:10.1186/s13063-024-08254-y)
Supplement: Supplementary file 1 — Supplementary Material 1: Supplemental Table S1. Complexity score. Supplemental Table S2. Statistical power to detect the given intervention effect 3 sample-size scenarios. Statistical power was estimated with Monte Carlo simulation assuming that the overall baseline percentage of patients received interpreter services is 15% and cluster specific percentages range from 10 to 20%. Supplemental Table S3. WHO Trial Registration Data Set information (Version 1.3.1). [file 13063_2024_8254_MOESM1_ESM.docx]

Supplemental table 1: Complexity score

| Variables | Severe Complexity  (3 points for each variable) | Moderate Complexity  (2 points for each variable) | Mild Complexity  (1 point for each variable) |
| --- | --- | --- | --- |
| Length of Stay | ≥ 7 days | 3- 6 days | <3 days |
| Level of Care | ICU | PCU/floor |  |
| Events (clinical notes from teams and services involved in care, procedural notes and diagnostic reports) | ≥ 8 on two consecutive days | 4-7 on two consecutive days | <=3 on two consecutive days |
| Palliative Care Score | Palliative Care Consultation or New Palliative Care Score (Complexity) ≥  70 | New Palliative Care Score (Complexity) 69-50 | <50 |

Supplemental table 2 Statistical power to detect the given intervention effect 3 sample-size scenarios **Legend:** Statistical power was estimated with Monte Carlo simulation assuming that the overall baseline percentage of patients received interpreter services is 15% and cluster specific percentages range from 10% to 20%

| Intervention | Statistical power | | |
| --- | --- | --- | --- |
| Odds ratio | 500 per cluster | 600 per cluster | 700 per cluster |
| 1.35 | 39% | 43% | 49% |
| 1.40 | 53% | 58% | 63% |
| 1.45 | 65% | 72% | 77% |
| 1.50 | 76% | 81% | 87% |
| 1.55 | 84% | 89% | 94% |
| 1.60 | 91% | 95% | 97% |
| 1.65 | 96% | 98% | 99% |

Supplemental table 3 WHO Trial Registration Data Set information (Version 1.3.1)

| WHO Trial Registration Data Set item (Version 1.3.1) | Information |
| --- | --- |
| 1. Primary Registry and Trial Identifying Number | ClinicalTrials.gov Identifier: NCT05860777 |
| 2. Date of Registration in Primary Registry | May 8, 2023 |
| 3. Secondary Identifying Numbers | IRB #22-002926 |
| 4. Source(s) of Monetary or Material Support | This work was supported by U.S. Department of Health and Human Services and U.S. Public Health Service Agency for Healthcare Research and Quality Grant R21 HS028475. |
| 5. Primary Sponsor | Mayo Clinic |
| 6. Secondary Sponsor(s) | NA |
| 7. Contact for Public Queries | Brian Pickering, MB, B.Ch |
| 8. Contact for Scientific Queries | Amelia Barwise, M.B., B.Ch., B.A.O., Ph.D.  [barwise.amelia@mayo.edu](mailto:barwise.amelia@mayo.edu) |
| 9. Public Title | Harnessing Health IT to Promote Equitable Care for Patients With Limited English Proficiency and Complex Care Needs |
| 10. Scientific Title | Harnessing Health Information Technology to Promote Equitable Care for Patients With Limited English Proficiency and Complex Care Needs |
| 11. Countries of Recruitment | United States |
| 12. Health Condition(s) or Problem(s) Studied | Artificial Intelligence impact on improving interpreter use among patients with language barriers and complex care needs |
| 13. Intervention(s) | Intervention Name: Health Information Technology to Promote Equitable Care for Patients With Limited English Proficiency and Complex Care Needs  Intervention Description: Complexity score and implementation: AI machine learning model to identify patients with complex care needs and implementation with active outreach to clinicians to offer interpreter services   This is a pragmatic clinical trial - a stepped wedge cluster randomized control trial.  Study arms: 1) No Intervention - Control. This is the control group who receive standard of care 2) Experimental – Intervention. This is the group in units who are identified via AI model and in which interpreter services are actively reaching out to clinicians |
| 14. Key Inclusion and Exclusion Criteria | Inclusion Criteria: Adults with limited English proficiency and requiring an interpreter and having complex medical care needs (includes admitted to the ICU, prolonged hospital stay, requiring palliative care). Have given research authorization.  Exclusion Criteria: - Do not require an interpreter  and <18 years old Sexes Eligible for Study: All  18 Years and older (Adult, Older Adult)  Accepts Healthy Volunteers ICMJE: No |
| 15. Study Type | Type of study (interventional or observational): Interventional  Method of allocation (randomized/non-randomized): Randomized  Masking (is masking used and, if so, who is masked): None (Open Label)  Assignment (single arm, parallel, crossover or factorial): clusters begin the study in the control condition, are randomly assigned to sequences, and cross-over to the intervention condition at pre-determined time points in a sequential, staggered fashion until all groups or clusters receive the intervention  Purpose: Health Services Research  Phase (if applicable): Not Applicable |
| 16. Date of First Enrollment | May 1, 2024 |
| 17. Sample Size | Sample Size consists of: 8400  Number of participants that the trial plans to enroll in total: 8400  Number of participants that the trial has enrolled: 1800 |
| 18. Recruitment Status | Recruitment status of this trial: Enrolling by invitation  Pending: NA  Recruiting: 1800  Suspended: NA  Complete: participants are no longer being recruited or enrolled: NA |
| 19. Primary Outcome(s) | Number of interpreter visits [ Time Frame: 12 months ]  The number of interpreters used among patients with LEP and complex care needs |
| 20. Key Secondary Outcomes | Time to interpreter visit [ Time Frame: 12 months ]  The time measured in hours and minutes for an interpreter to be used for patients with LEP |
| 21. Ethics Review | Mayo Clinic Institutional Review Board - ID: 22-002926  Submitted: 3/18/2022 4:09 PM |
| 22. Completion date | Anticipated June 21, 2024 |
| 23. Summary Results | NA |
| 24. IPD sharing statement | Plan to Share Individual Participant Data (IPD): No |
